# Editorial: Molecular Characterization of Humic Substances and Regulatory Processes Activated in Plants

**DOI:** 10.3389/fpls.2022.851451

**Published:** 2022-02-09

**Authors:** Serenella Nardi, Michela Schiavon, Adele Muscolo, Diego Pizzeghello, Andrea Ertani, Luciano Pasqualoto Canellas, Jose M. Garcia-Mina

**Affiliations:** ^1^DAFNAE, Università degli Studi di Padova, Padua, Italy; ^2^DISAFA, University of Torino, Turin, Italy; ^3^Department of AGRARIA, “Mediterranea” University, Reggio Calabria, Italy; ^4^Núcleo de Desenvolvimento de Insumos Biológicos para Agricultura (NUDIBA), Universidade Estadual do Norte Fluminense Darcy Ribeiro (UENF), Rio de Janeiro, Brazil; ^5^Department of Environmental Biology, Sciences School, University of Navarra, Pamplona, Spain

**Keywords:** humic substances (HS), biostimulants, microbiome, soil quality (SQ), plant nutrition and metabolism

The intense crop fertilization along with to climate change have a negative synergistic impact on soil fertility by decreasing the soil organic matter content and biological activity. As a result of these processes, plant productivity, and quality could be harshly impaired in agroecosystems. This is of global concern, particularly considering that a world with zero hunger is a challenge set in the 2030 Agenda for Sustainable Development, and that the COVID-19 pandemic has aggravated the condition of vulnerable populations. In this context, green and sustainable strategies could be endorsed to boost crop productivity and improve soil fertility. In particular, the role of humic substances (HS) as activators of plant development and metabolism, as well as stimulators of beneficial rhizosphere microorganisms attains great attention. Thus, the present topic collection encompasses studies that investigate HS action in either small-scale or agronomic trials.

One area of research is the link between structure of HS and their activity. Monda et al. studied the biostimulant properties of a sedimentary shale ore-extracted humic acid (HA) on tomato plants grown under increasing nutritional stress. The authors investigated the chemical features of HA and found that HA alleviated the nutritional stress of plants by improving their nutrient use efficiency more than plants fertilized with high NPK. Yield and fruit quality were enhanced by HA. Pizzeghello et al. showed that treating HS with a weak acid generated low and high sized-fractionated HS with greater bioactivity, because of novel molecular arrangements of HS components that better interacted with roots. Also, the the cover vegetation of the soils from where HS were obtained affected their bioactivity. HS were applied to garlic and stimulated plant growth and nutrition. Lamar et al. studied the effects of seven ore-derived HA on maize. Chemometric analyses evidenced that the primary driver of plant biomass and morphology was the ratio between HA electron accepting capacity (EAC) and electron donating capacity (EDC). The HA EAC was due to quinones and semiquinone free radicals, while the HA EDC was ascribed to polyphenolics and glycosylated polyphenolics. From this manuscript emerges a mechanism of action for ore-derived HA biostimulation that involves the interplay of pro-oxidants and antioxidants. This manuscript also indicates how the EAC/EDC ratio can be adjusted to a proportion to produce seedlings with desirable qualities, providing evidence of methods to produce more efficient HAs.

Several studies have shown the role of the endophytic microbiome in the evolvement of relevant metabolic effects within the plant. In this framework is plausible that the mechanisms responsible for the beneficial action of HS on plant growth involve some previous action on the endophytic microbiome. The results presented by Della Lucia, Bertoldo, Broccanello et al. strongly support this hypothesis. The authors observed that the foliar application of a formulation obtained from leonardite on sugar beet plants cultivated either in hydroponics or open field, caused the increase of a specific family of endophytes, *Oxalicibacterium* spp., that has a known plant growth promoting effect. These results were associated with the upregulation of genes involved in the auxin-dependent signaling pathways and yield increases. The results reported by De Hita, Fuentes, Fernández et al. also support a role of endophytic microbiome in the mechanisms responsible for the humic acid beneficial action on plant growth, since many of the culturable endophytes activated by the application of a leonardite humic acid in cucumber have relevant plant biostimulant traits such as the biosynthesis of cytokinins and auxins, the production of organic acids and siderophores that are involved in iron and phosphorous mobilization in soil, as well as the ability to grow without nitrogen available in the nutrient media.

Galambos et al. investigated the growth-related processes, bacterial colonization, and transcriptional responses activated by the combined applications of endophytic bacterial strains and HA in tomato roots and shoots, and indicates the optimization of dosages, complementation properties, and gene markers for the production of efficient PGPB- and HA-based biostimulants.

Della Lucia, Baghdadi, Mangione et al. described the effects of a biostimulant on tomato plants grown in well-watered and drought conditions. The biostimulant applied to roots increased the photosynthetic rate and the chlorophyll content of plants under drought, compared to the standard fertilizer, led to higher fruit dry matter and reduction in the number of cracked fruits, and improved the resistance of tomato to drought. De Hita, Fuentes, Zamarreño et al. evaluated the mechanisms of action of foliar application vs. root application of a sedimentary humic acid (SHA). Six markers related to plant phenotype, plant morphology, hormonal balance and root-plasma membrane H^+^- ATPase were studied. Both application methods improved the plant growth, the concentrations of jasmonic acid and jasmonoyl-isoleucine and indole-3-acetic acid in roots and cytokinins in shoots. Foliar application did not lead to short-term increases in abscisic acid root-concentration and root plasma membrane H^+^-ATPase activity, which were instead triggered by SHA root-application. This study suggest that the beneficial effects of SHA may result from plant adaptation to a mild transient stress caused by SHA application.

Different studies also evidenced the efficacy of HS as possible amendments, but few field researches considered the environmental factors of HS efficacy. Olk, Dinnes, Scoresby et al. evaluated the spatial and temporal variability in the efficacy of a micronized humic product on maize growth and grain yield in two rainfed fields supporting a maize–soybean rotation. Application of the humic product during four maize seasons evidenced that grain quality remained unchanged. Protein, starch, nutrient, and oil content showed only few significant responses to humic product application, while increases in agronomical maize traits were observed.

Field evaluations of commercial humic products have seldom involved replication across location or year. To evaluate the consistency of HS efficacy in field conditions, by Olk, Dinnes, Callaway et al. determined the effects of a humic product on maize growth in high-yielding Midwestern fields through replicated strip plots in five site-year combinations, and through demonstration strips in 30–35 production fields annually for 2009–2011 that covered major areas of Iowa. Olk, Dinnes, Callaway et al. demonstrated the capability of a humic product to improve maize growth in high-yielding conditions. Humic product application increased total leaf area in all field treatments at three site-year combinations.

In another field study, Vujinovic et al. studied the bioactivity of dissolved HS (DHS), isolated from the conversion of conventional (CF) farming and organic (OF) farming soil leachates, in maize. DHS were collected from bare and planted soils and stimulated lateral roots proliferation, nitrate uptake, and modulated genes involved in nitrogen acquisition. Wheat roots in soil, in particular, boosted the rhizosphere biological activity, and mineralization processes. The authors demonstrated that OF and CF managements of soil influenced the characteristics of DHS and that plant roots can interact with the active molecules in the soil solution.

## Author Contributions

All authors contributed to the article and approved the submitted version.

## Conflict of Interest

The authors declare that the research was conducted in the absence of any commercial or financial relationships that could be construed as a potential conflict of interest.

## Publisher's Note

All claims expressed in this article are solely those of the authors and do not necessarily represent those of their affiliated organizations, or those of the publisher, the editors and the reviewers. Any product that may be evaluated in this article, or claim that may be made by its manufacturer, is not guaranteed or endorsed by the publisher.

